# Impact of COVID-19 on emergency department visits for individuals with psychotic disorders in Newfoundland and Labrador, Canada: A longitudinal study

**DOI:** 10.1371/journal.pgph.0004836

**Published:** 2025-08-07

**Authors:** Zachary E. M. Giovannini-Green, Gerald Mugford, Zhiwei Gao

**Affiliations:** Discipline of Clinical Epidemiology, Division of Population Health and Applied Health Sciences, Memorial University of Newfoundland and Labrador, St. John’s, Newfoundland and Labrador, Canada; University of Tunis El Manar Faculty of Medicine of Tunis: Universite de Tunis El Manar Faculte de Medecine de Tunis, TUNISIA

## Abstract

During the global COVID-19 pandemic, emergency departments (EDs) saw an overall decrease in utilization. However, some vulnerable groups, such as those living with psychotic disorders, must often rely on the services provided by EDs. The literature in this area lacks methodologically robust longitudinal studies which examine the ED utilization patterns of individuals with psychotic disorders both before and during the pandemic. This research was a longitudinal retrospective cohort study of individuals with a psychotic disorder in Newfoundland and Labrador before COVID-19 (2011–2019) and during the pandemic (2020–2022). Patients diagnosed with a psychotic disorder between April 1^st^, 2011, and March 31^st^, 2022, who were between 15–24 years old, and who visited an ED at least once, were included. Average monthly visits were used to measure ED visits to control for differences in each individual’s data. Independent variables in the analyses were: 1) age, 2) sex, 3) geographic region, 4) urban or rural residence, and 5) ED visit before or during COVID-19. Multiple linear regression with Generalized Estimating Equations (GEE) modelling was used to identify factors associated with mean monthly ED visits. Multivariate analysis showed the mean monthly ED visits increased significantly during the first year of COVID-19 than before the pandemic (Mean = 0.30 vs Mean = 0.21, p = 0.01). Female individuals and rural residents also had significantly higher mean monthly ED visits than male individuals (Female = 0.51 vs Male = 0.21, p < 0.01) and urban residents (rural = 0.38 vs urban = 0.21, p = 0.02). ED utilization by individuals with psychotic disorders increased during the first year of the COVID-19 pandemic, while ED use by most Canadians decreased. This study identified the demographics of Canadians who require extra support during a health crisis. There is always the possibility of another global pandemic, and the Canadian healthcare system must be equipped to provide adequate services to all Canadians, especially those who are most marginalized.

## Introduction

Approximately 18% of all Canadians will meet the criteria for at least one mental disorder each year [1]. Despite this, in 2020, Canadians waited an average of 22 days to receive potentially life-saving community mental health counselling [2]. This results in the Canadians having to resort to accessing emergency departments (EDs) to receive some form of care. Indeed, approximately ten percent of Canadians who visit an ED with symptoms of mental or substance use disorder will access an ED four or more times every year [3]. Those living with severe psychotic disorders, such as schizophrenia, are particularly likely to have multiple visits to an ED each year [4,5]. Starting in 2020, the COVID-19 pandemic caused mass disruptions to the healthcare system as nearly all resources had to be suddenly allocated to the management of the virus.

This resulted in a widely reported overall drop in visits to emergency departments in Canada [6,7] and in other countries, including Israel, Finland, the Netherlands, the United States, and the United Kingdom [8–12], especially during the first year of the pandemic. This trend continued with overall presentations at EDs for psychiatric admissions in general, as seen globally in countries such as Australia, France, Italy, Turkey, and New Zealand [13–17]. However, an increase in presentations to EDs of individuals with symptoms of psychosis and psychotic disorders during the COVID-19 pandemic has been reported by several studies conducted in countries outside of Canada, with the majority conducted in Europe [13,18,19].

In Newfoundland and Labrador, there has been a rise in concern about mental health, as seen in a 2022 document published by the provincial government [20]. Newfoundlanders and Labradorians have had to wait on average longer than other Canadians for health services. As an example of this, in 2020, the average wait time for community mental health counselling was 33 days, 11 days longer than the national average [2]. Of special concern is the mental health of the youth in the province. The average age of onset of schizophrenia and related disorders has recently been reported as approximately 20 years of age according to a large-scale meta-analysis involving 192 studies [21]. This adds another level of stress to young people during an already difficult stage in their lives. It also affects the loved ones of those affected. The onset of a psychotic disorder can cause youth to display irritation and anxiety, withdraw from their social lives, and develop bizarre thoughts and ideas [22]. Stigma from the perspective of the individual, both from others and from themselves, as well as a lack of knowledge about mental health, has also been shown to delay accessing treatment and prolong the duration of untreated psychosis [23]. The duration of untreated psychosis (DUP) is known to be negatively correlated with outcomes for those who experience psychotic episodes [24,25].

These factors affect the likelihood of youth actively seeking help for their mental health. In Canada, less than a quarter of youth will seek help of any kind with their mental health, and often from online resources as opposed to mental health professionals [26,27]. This is despite Canadian research that recommends increased mental health services for youth, given the rise in prevalence of these illnesses in recent years [28]. This may result in youth not accessing services until their symptoms become acute, resulting in their necessary utilization of EDs. Despite the effect of this on youth, no literature exists that examines the effects of the COVID-19 pandemic on youth living with psychotic disorders in Newfoundland and Labrador.

The global decrease in utilization of ED services during the COVID-19 pandemic is well documented in the literature. The increase in the utilization of individuals presenting with psychosis symptoms has also been previously studied. However, most studies conducted on this topic have used European data. Furthermore, most have been cross-sectional with limited follow-up. Comparisons to pre-pandemic utilization, therefore, use different individuals, introducing a source of confounding. A longitudinal study is needed to investigate the overall trend of utilization of EDs by individuals with diagnosed psychotic disorders during the pandemic in comparison with their pre-pandemic levels of utilization. A study that investigates this issue from the Canadian perspective would also add to the literature.

### Research objective

This research will answer the following question: Is there a difference in the trend of the number of all ED visits for individuals diagnosed with a psychotic disorder before the pandemic (2011–2019) and during the COVID-19 pandemic (2020–2022) when examined longitudinally?

## Methods

### Study population

Administrative databases maintained by Digital Health NL for the Canadian province of Newfoundland and Labrador were utilized for this retrospective cohort study. Study analysis included individuals diagnosed with a psychotic disorder between April 1^st^, 2011, to March 31^st^, 2022, and aged 15–24 who accessed an ED at least once. For this study, schizophrenia and schizoaffective disorder were the two psychotic disorders included. Individual diagnoses in the administrative database were identified using ICD-10-CA diagnostic codes. This study has received approval by the Health Research Ethics Authority of Newfoundland and Labrador (HREB #2022.214).

### Data sources

Data relating to ED utilization was collected using the MediTech Data – Emergency Department Module, which contains demographic, clinical, and administrative data, including date and time of arrival and discharge, as well as presenting complaint. The provincial Client Registry (CR) was accessed for demographic information from individuals presenting at a hospital or pharmacy. The Provincial Discharge Abstract Database (PDAD) provided the demographic, clinical, and administrative data of individuals upon discharge from inpatient services or surgical day care services. The Master Geography File (MGF) determined in which of the four regional health authorities (RHAs) an individual resided. This was done by comparing the six-digit postal code to Statistics Canada’s standard geographical areas. To determine urban or rural residence, the number character in the first three digits of the postal code was used [29]. Digital Health NL provided the data linkage and created a unique and de-identified patient number to facilitate the linkage of the data from the above databases. The authors submitted a data access request to Digital Health NL and, upon receiving the de-identified data, created a single database which included all relevant variables for eligible individuals. This constituted the study sample for this research.

### Measuring utilization of emergency departments

The difference in monthly utilization of EDs during and before the COVID-19 pandemic was the variable being investigated for this study. For each individual, four means were calculated. The first was the mean monthly visits before the COVID-19 pandemic, which in this study was taken as April 1^st^, 2011, to December 31^st^, 2019. The second and third were the mean monthly visits during the calendar years of 2020 and 2021, respectively. The fourth was the mean monthly visits during the first three months of 2022, from January 1^st^ to March 31st. To calculate the means, the total number of ED visits was divided by the number of months in the period. This amounted to 105 months in the first period, twelve months in the second and third periods, and three months in the fourth period. All visits to an ED were included in this study to obtain a full understanding of how individuals diagnosed with psychotic disorders utilize EDs in this province.

The independent variables included in the univariate and multivariate analyses will consist of the following: 1) age at diagnosis 2) sex 3) patient regional health authority (RHA), 4) urban or rural residence as determined by Digital Health NL using the MGF and 5) time periods as outlined in the above section.

### Statistical analysis

Descriptive statistics were generated to describe the study population, including counts and proportions, and means and standard deviations (SD) for categorical and continuous variables, respectively. Multiple linear regression with Generalized Estimating Equations (GEE) modelling was used to identify the significant factors associated with mean monthly ED visits in both the univariate and multivariate analyses. Autoregression was selected as the working correlation matrix to meet the needs of the longitudinal nature of the study design. All analyses were conducted using SAS 9.4.

## Results

### Overview

Between April 1^st^, 2011, and March 31^st^, 2022, 125 individuals diagnosed with a psychotic disorder accessed an ED in the province at least once. Out of the 125 individuals, 94 (75.2%) were male, 83 (66.4%) resided in urban areas, and 66 (52.8%) lived in the Eastern Health RHA. The mean monthly ED visits before the COVID-19 pandemic was 0.20 (SD: 0.18), rising during the first year of the pandemic (0.30, SD: 0.47), and then returning to levels similar to before the pandemic. A full overview is available in Table 1.

**Table 1:** Overview of individuals in the study.

Variable (N = 125)	Descriptives
**Sex, N(%)**	
Female	31 (24.8%)
Male	94 (75.2%)
**RHA, N(%)**	
Central	8 (6.4%)
Western	25 (20.0%)
Labrador-Grenfell	26 (20.8%)
Eastern	66 (52.8%)
**Urban or Rural Residence, N(%)**	
Rural	41 (32.8%)
Urban	83 (66.4%)
**Age at Diagnosis, mean (SD)**	20.64 (2.40)
**Mean Monthly ED Visits, mean (SD)**	
Before COVID	0.20 (0.18)
First Year of COVID	0.30 (0.47)
Second Year of COVID	0.23 (0.42)
Third Year of COVID	0.23 (0.66)

### Effect of COVID-19 on emergency department visits

In the univariate analysis, during the first year of the pandemic, there was a statistically significant increase in the mean monthly number of emergency department visits over the pre-pandemic figure (B-Coefficient = 0.10, p = 0.01). The mean monthly number of ED visits then returned to levels similar to the pre-pandemic figure for the rest of the study period. The period variable, however, was not statistically significant (p = 0.07). Female sex was significantly associated with an increase in mean monthly ED visits (B-Coefficient = 0.27, p < 0.01). Residence in a rural area was shown to predict an increase in the mean monthly ED visits (B-Coefficient = 0.14, p = 0.06), although it is borderline significant. No other variables were significant predictors in the univariate analysis. A full description of the results of the univariate analyses is available in Table 2.

**Table 2:** Univariate analyses.

Variable	B-Coefficient	Standard Error	p-Value
**Sex**			
Female	0.27	0.09	<0.01*
Male	0		
**Age at Diagnosis**	−0.01	0.01	0.58
**RHA**			
Central	0.14	0.17	0.40
Western	−0.01	0.04	0.84
Labrador-Grenfell	0.17	0.11	0.10
Eastern	0		
**Urban or Rural Residence**			
Rural	0.14	0.07	0.06
Urban	0		
**Mean Monthly ED Visits**			
First Year of COVID	0.10	0.04	0.01*
Second Year of COVID	0.03	0.04	0.40
Third Year of COVID	0.03	0.06	0.64
Before COVID	0		

Where * indicates a statistically significant value.

In the multivariate analysis, the effect of the COVID-19 pandemic on mean monthly ED visits for individuals in the study was similar to that shown in the univariate analysis. The increase in ED utilization for the first year of the pandemic was statistically significant (B-Coefficient = 0.09, p = 0.01), and returned to levels similar to pre-pandemic for the second and third years of the COVID-19 pandemic. Female sex and rural residence also predicted a statistically significant higher mean monthly ED visits (B-Coefficient = 0.30, p < 0.01; B-Coefficient = 0.17, p = 0.02). RHA was not significant in predicting mean monthly ED visits and so was removed from the multivariate analysis after all interaction terms were analyzed. A full description of the results of the multivariate analyses is available in Table 3.

**Table 3:** Multivariate analyses.

Variable	B-Coefficient	Standard Error	p-Value
**Sex**			
Female	0.30	0.09	<0.01*
Male	0		
**Age at Diagnosis**	−0.01	0.01	0.58
**Urban or Rural Residence**			
Rural	0.17	0.07	0.02*
Urban	0		
**Mean Monthly ED Visits**			
First Year of COVID	0.09	0.04	0.01*
Second Year of COVID	0.03	0.04	0.39
Third Year of COVID	0.03	0.06	0.66
Before COVID	0		

Where * indicates a statistically significant value.

## Discussion

### COVID-19 and emergency department visits

This study indicated that the utilization of EDs by those diagnosed with schizophrenia and schizoaffective disorder increased significantly during the first months of the COVID-19 pandemic but then returned to levels similar to pre-pandemic for the rest of the study period. These results are consistent with previous research in other countries [13,18,19]. These results differed from the trend of ED visits for all psychiatric disorders [13–17], which decreased during the same period. Given the age criteria of the study, the high percentage of males in the study is consistent with research indicating that males have an earlier average age of onset relative to females [30]. The percentage of individuals living in urban areas and in the Eastern Health RHA is consistent with percentages in the general population. Within Canada, the increase in ED utilization for individuals with schizophrenia and schizoaffective disorder may be at least partially explained by research performed by Stephenson et al. [31]. This study found significant disruption to all services provided to individuals living with schizophrenia during the pandemic in comparison with pre-pandemic services.

The significant increase in mean monthly ED visits for individuals with schizophrenia and schizoaffective disorder demonstrated by this study should be compared to the decrease in ED utilization for all other complaints. This contradiction suggests that those living with severe and persistent psychotic disorders are more vulnerable than those with other types of disorders during a pandemic. It also indicates that vital services for these individuals are being compromised, posing serious risks to their recovery and overall health and well-being. This has been confirmed in other parts of Canada by the research of Stephenson et al. [31]. The idea that those living with severe and persistent psychotic disorders are highly vulnerable to stress is not a new concept. This line of inquiry began with the oft-cited work of Zubin and Spring [32] in the 1970s. In the intervening decades, there have been continued efforts to further examine the correlation between stress and psychosis from a neurobiological perspective [33,34]. What should also be mentioned here is the decrease in ED utilization after the first year of the pandemic. This is indicative of two factors, either separate or in combination: 1) the ability of individuals in the study to adapt to the situation that the pandemic has created, and 2) the resumption of services which had been temporarily halted during the first year of the pandemic. Regardless, the sharp increase in utilization during the initial stages of the pandemic is a lesson and a reminder of the effect that this type of crisis has on the most vulnerable in our population.

This study adds to the literature on this topic by highlighting the role of sex and urbanicity as factors that predicted increased utilization of EDs by women and rural-dwelling individuals during the COVID-19 pandemic. The results of this study are consistent with research findings that females had higher ED utilization rates than males during the pandemic, particularly for mental health-related visits [35,36]. This may be attributed to the fact that females were shown to be more often put into the role of caregiver during COVID-19 than men, and their perceived level of mental health was lower [37,38]. Both of these factors are likely to increase the likelihood of women requiring the services of an ED.

Research on psychotic disorders in rural areas focuses heavily on the Global East and South, with few instances of research in North America [39–41]. This is likely because urban dwelling has been repeatedly identified as a risk factor for psychosis [42–44]. Nevertheless, the risk of increased utilization of EDs in rural areas during the COVID-19 pandemic can be explained by two significant factors. One, rural populations are underserved by medical professionals, with only 12.8% of family physicians and 2.2% of specialists serving approximately 18% of Canadians [45,46]. Additionally, approximately 30% of people in Newfoundland and Labrador do not have regular access to a family doctor [47,48]. Two, early intervention services for psychosis in Newfoundland and Labrador are largely available only to urban-dwelling youth and are provided by programs such as the psychosis early intervention and recovery program [49]. Three, individuals living in rural communities struggle to access medical treatment, and when they do, their outcomes are poorer [50,51]. When taken together, this creates a situation whereby those living in rural areas must rely on EDs for their critical care.

### Study strengths and limitations

This study has several strengths that address the literature gap as stated in the introduction. First, this study highlights the reality of those living with psychotic disorders during the COVID-19 pandemic and how their patterns of use for EDs differ from the general population. Second, this study differs from the majority of studies on this topic in its use of a longitudinal methodology, which studies the same individual across the study period. This removes the potential bias of using different cohorts for the pre-pandemic and pandemic analyses and strengthens the validity of the results. Finally, this study identifies female sex and rural residence as factors that are significantly associated with an increased utilization of EDs in this population. These results are of value when developing policies that deliver strategic support to these individuals.

This study was limited to individuals living in Newfoundland and Labrador who were:

1) diagnosed between the ages of 15–24 with schizophrenia or schizoaffective disorder, and 2) who accessed an ED at least once during the study period. This research, therefore, did not include those who accessed an ED with symptoms of psychosis but without a diagnosis of the above disorders. It also did not include those with a diagnosis that was made before the study period, but who accessed an ED. Those who were diagnosed with schizophrenia or schizoaffective disorder after the age of 24 were similarly not included. Individuals aged 15–24 constitute the demographic most affected by psychotic disorders, as shown by research linking earlier age of onset to poorer overall outcomes, regardless of sex [52]. Therefore, while research investigating individuals diagnosed after age 24 is a legitimate line of inquiry, the most vulnerable individuals affected by psychotic disorders were the focus of this study. The data provided did not identify the reason for visiting the ED. Only the year of visit to an ED was available, and not day and month, making the analysis of monthly or seasonal trends impossible. Finally, this study was conducted using a study population from Newfoundland and Labrador only. Therefore, generalizing these results to other regions should be done with appropriate caution.

### Further research

Further research on this topic may be conducted to investigate how the COVID-19 pandemic affected the utilization of youth, specifically those who had pre-existing diagnoses of psychotic disorders. These results may then be compared with individuals who first presented with symptoms of psychosis during the COVID-19 pandemic to find any differences that appear in utilization patterns. Research into trend analysis for the months and seasons during the pandemic may also provide critical information that helps clinicians and policymakers plan the staff and technical resources that will be required to meet the needs of all individuals. This would not be limited to a retrospective analysis of the COVID-19 pandemic. The changing nature of population health is such that new viruses and their subsequent pandemics are a constant possibility and should be prepared for by public health groups. The clade I strain of mpox (previously known as monkeypox) is the most recent virus to gain international attention. In August 2024, it was declared by the World Health Organisation as a public health emergency of international concern [53].

## Conclusion

This study was conceptualized to investigate how people with diagnosed severe psychotic disorders utilized EDs both before and during the COVID-19 pandemic. The results of this study identified three variables that significantly predicted utilization for individuals with severe psychotic disorders accessing EDs before and during the COVID-19 pandemic. First, the utilization of EDs increased significantly during the first year of the pandemic and then returned to levels of utilization similar to those seen pre-pandemic. Second, females were significantly more likely than males to have increased utilization of the EDs during the pandemic. Third, rural residence predicted increased utilization of EDs to a level that approached significance. Total utilization of EDs has been shown to have decreased in Canada during the first year of the pandemic [6,7]. However, those individuals with severe psychotic disorders accessed those services at a time when other Canadians were staying away from hospital EDs. This serves as a reminder of how the most vulnerable and underserved people in our society access care differently from the rest of the population. It is also a reminder that we have a responsibility to provide adequate supports to them so that they are able to have the best possible health outcomes.
